# Severe Babesiosis Masquerading as Thrombotic Thrombocytopenic Purpura: A Case Report

**DOI:** 10.7759/cureus.4459

**Published:** 2019-04-15

**Authors:** Daniel Chang, Md Hossain, Mohammed A Hossain

**Affiliations:** 1 Nephrology, New York-Presbyterian Queens, Flushing, USA; 2 Internal Medicine, Jamaica Hospital Medical Center, Jamaica, USA

**Keywords:** babesiosis, microangiopathic hemolytic anemia

## Abstract

Babesiosis is an infectious disease caused by protozoa of the genus Babesia. Its incidence has been increasing in the Northeast and upper Midwest regions of the United States in recent years. Symptoms of babesiosis can range from a mild flu-like illness to acute, severe, and fatal disease. Severe disease is common in older populations and immunocompromised patients. The laboratory abnormalities associated with babesiosis, such as low hematocrit, low hemoglobin, elevated lactate dehydrogenase, low haptoglobin, reticulocytosis, the presence of schistocytes on the peripheral blood smear and thrombocytopenia, can mimic other severe systemic disorders like thrombotic thrombocytopenic purpura (TTP). Hence, it is crucial to be aware of babesiosis, especially in highly endemic areas as it can masquerade as severe fatal systemic diseases like TTP.

## Introduction

Microangiopathic hemolytic anemia (MAHA) is a non-immune intravascular hemolytic anemia characterized by the presence of schistocytes on peripheral blood smear [1]. Thrombotic thrombocytopenic purpura (TTP), a medical emergency and an almost uniformly fatal disease without appropriate treatment, can present with MAHA [2]. However, many different disorders, including systemic infections, malignancies, adverse drug reactions, severe hypertension, preeclampsia, systemic lupus erythematosus (SLE), allogeneic hematopoietic stem cell transplantation, and abnormalities of complement regulation also present with MAHA. Hence, it is essential to be aware of the etiologies of MAHA for appropriate and prompt treatment [3]. This article presents a case of human babesiosis presenting with MAHA and thrombocytopenia.

As the incidence of Babesia infection has been increasing across North America in recent years and as its clinical presentation of microangiopathic hemolytic anemia (MAHA) with thrombocytopenia is similar to other severe systemic disorders, it is crucial to be able to recognize all spectrums of babesiosis including the possibility of coinfection with other tick-borne diseases to prevent delays in diagnosis and treatment.

## Case presentation

**Figure 1:**
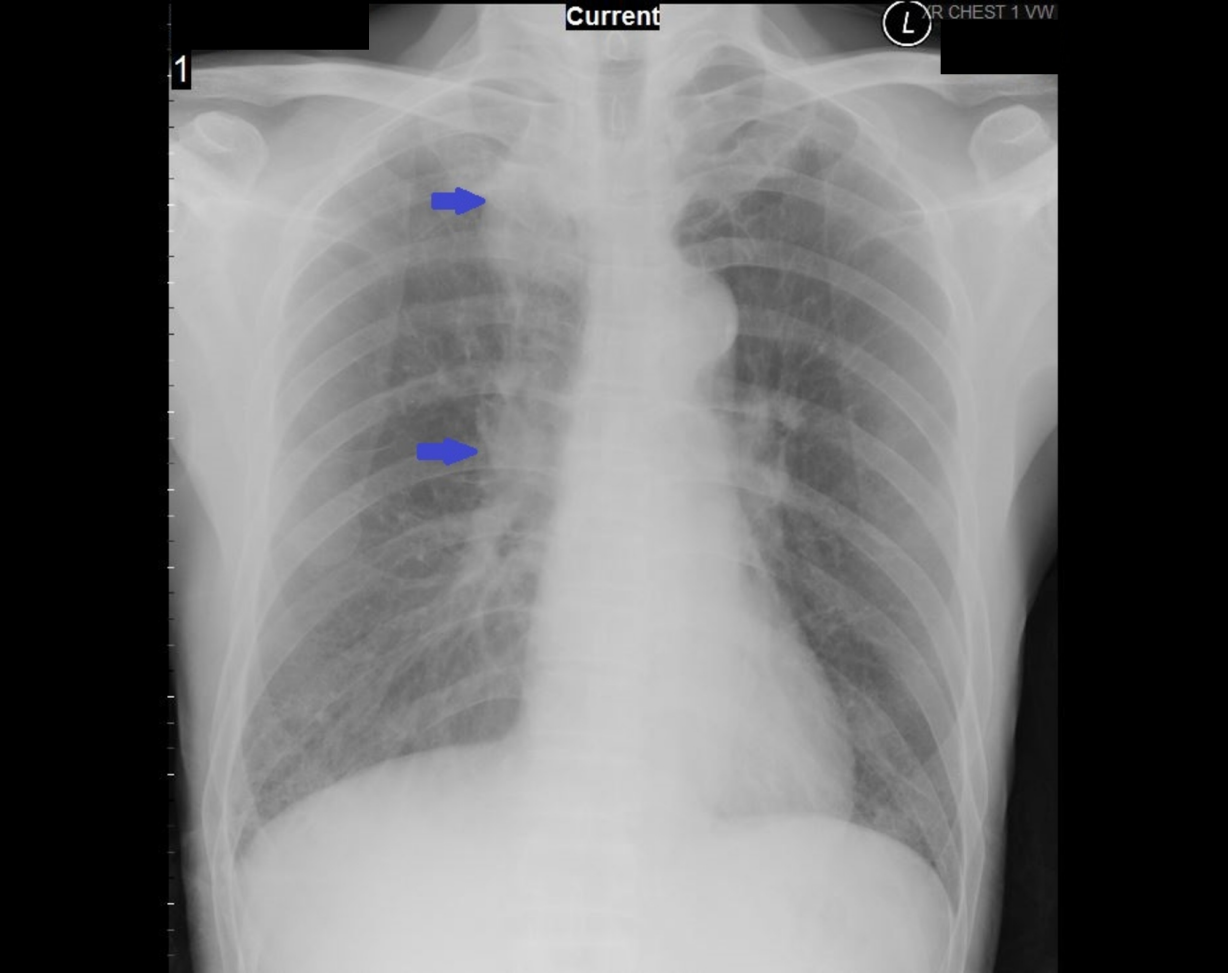
The chest X-ray showing postoperative changes of the right lung, pulmonary fibrosis, and borderline enlarged lower mediastinal lymph nodes adjacent to the distal esophagus.

In August 2018, a 69-year-old Asian male presented to the emergency department for five days of subjective fever with chills and generalized weakness. Three days before presentation, he had been prescribed a course of amoxicillin-clavulanic acid for possible pneumonia. His past medical history was remarkable for right upper lobe lung cancer that is currently in remission after being treated with lobectomy in 2012 and adjuvant chemotherapy completed five years ago, hypertension controlled with daily atenolol 25 mg, hepatitis B carrier on daily tenofovir 300 mg, nephrolithiasis status post lithotripsy, chronic kidney disease, and benign prostatic hyperplasia. A recent outpatient chest X-ray showed postoperative changes of the right lung, pulmonary fibrosis, and borderline enlarged lower mediastinal lymph nodes adjacent to the distal esophagus (Figure 1). His outpatient blood tests were significant for transaminitis and platelet count of 42 k/μL. The patient denied any recent travel history or tick bites but stated that he had a golfing trip in Westchester, New York until one day before feeling sick. His social history was only remarkable for drinking alcohol. He quitted smoking for two months and denied any illicit drug use.

In the emergency department, he was febrile (temperature of 39.7°C), tachycardic (77 - 122 bpm), tachypneic (15 - 26 breath per minutes) and hypotensive (85/39 mmHg - 118/69 mmHg). His blood pressure was responsive to 3 liters of the normal saline bolus. He was awake and oriented. His physical examination was unremarkable except for bilateral mild yellow tint conjunctiva. His blood tests showed anemia (Hgb 8.7 g/dL; Hct 26.4%), neutropenia (WBC 4.71 K/μL), thrombocytopenia (platelet 37 K/μL) and low haptoglobin (<10 mg/dL). Our patient received one dose of intravenous (IV) piperacillin-tazobactam for presumed sepsis.

Three blood cultures were collected and they later showed no bacterial growth. Urinalysis detected few bacteria, moderate blood, urine protein of 30 mg/dL and urine urobilinogen of 4.0. His liver function tests showed elevation in total bilirubin of 3.5 mg/dL with direct bilirubin of 1.6 mg/dL and indirect bilirubin of 1.9 mg/dL, and lactate dehydrogenase of 636 U/L. His serum chemistry results were remarkable for serum sodium of 131 mmol/L, serum carbon dioxide of 17 mmol/L, blood urea nitrogen of 29.0 mg/dL, and serum creatinine of 1.39 mg/dL. Immunological tests including C3, C4, myeloperoxidase Ab, protease 3 Ab, and ANA were not significant except that the C3 level was 55 mg/dL.

**Figure 2:**
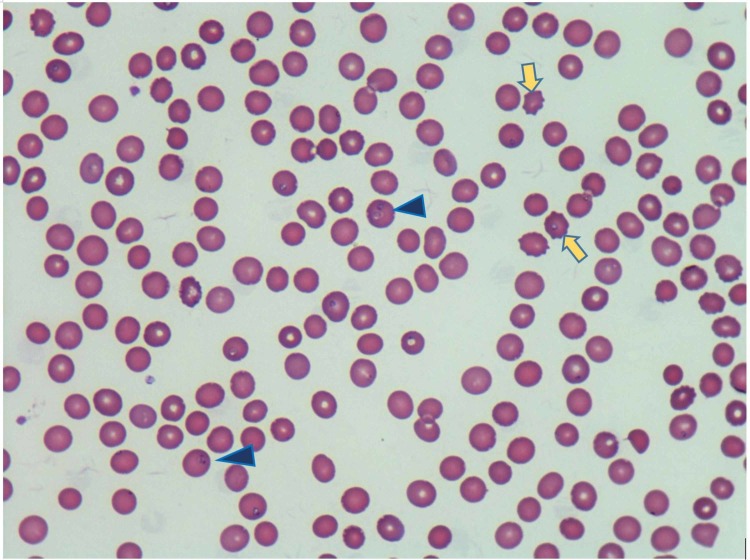
Wright-Giemsa stained blood smear under the 60x objective showing Babesia parasites in red blood cells (blue triangle arrows), Burr cells (yellow arrows), few polychromasia, slight anisocytosis, and moderate poikilocytosis.

**Figure 3:**
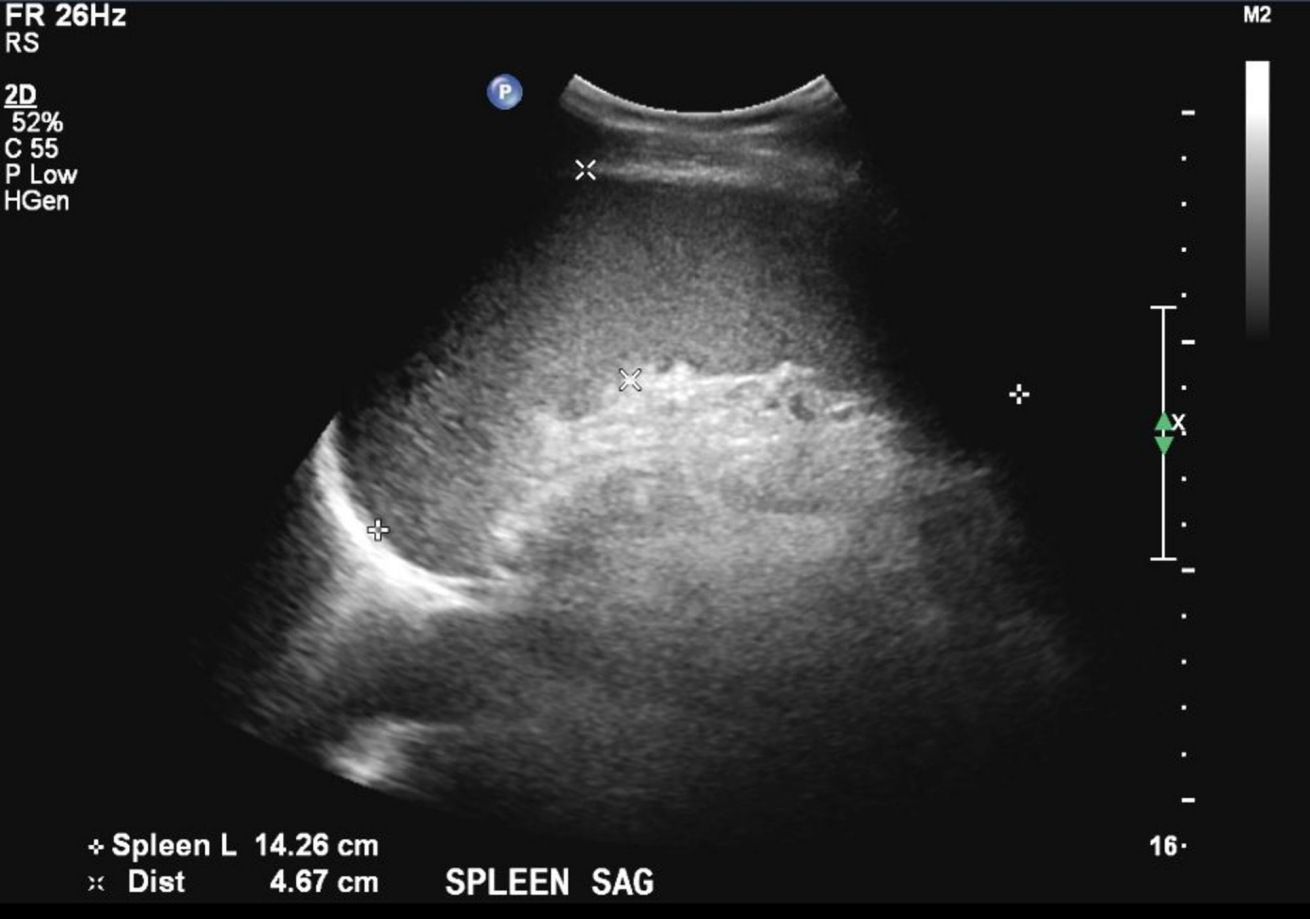
Abdominal ultrasound showing mild splenomegaly.

**Figure 4:**
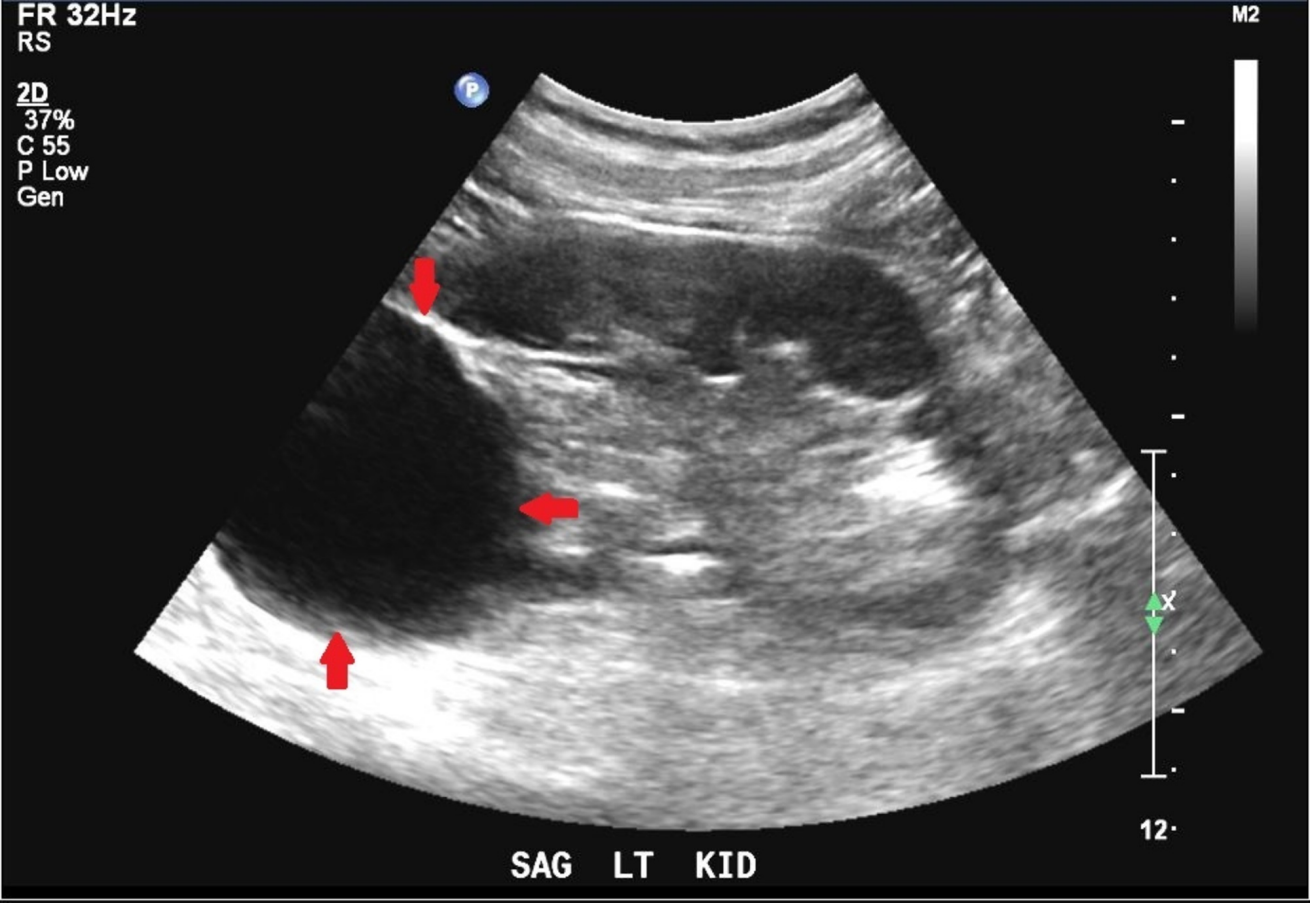
Abdominal ultrasound showing a 6.2 cm cyst at the upper pole of the left kidney.

A blood smear showed slight anisocytosis, moderate poikilocytosis, moderate Burr cell, and few polychromasia, but no schistocytes. It also revealed 25% of atypical lymphocytes with some toxic granulation and possible parasites in RBCs (Figure 2). Imaging studies were remarkable for mild splenomegaly (Figure 3) and left renal cyst (Figure 4).

Empirical treatment for parasitic infection to cover babesiosis and Lyme disease was started with Clindamycin 600 mg PO q8h, quinine 650 mg PO q8h, and doxycycline 100 mg PO BID as there was a strong concern for a parasitic infection as noted on the peripheral smear. Lyme IgG and IgM Western blot serology, Anaplasma Qualitative Real-time PCR and Babesia DNA Real-time PCR were performed to confirm the diagnosis and only the Babesia test came back as positive. Our patient responded well to the treatment regimen, and his hemoglobin and platelet count were 9.1 g/dL and 158 k/μL, respectively, at the time of hospital discharge. Oral Clindamycin, quinine, and doxycycline were continued to complete a 10-day course.

## Discussion

Before the role of autoantibody to ADAMTS13 was recognized, clinicians considered the presence of thrombocytopenia and microangiopathic hemolytic anemia even in the absence of overt organ dysfunction to initiate plasma exchange for TTP management [4]. Initial plasma exchange treatment is crucial to prevent potentially life-threatening complications of TTP. We therefore initially treated our patient for possible TTP. Finally, when we observed the blood smear suggestive of possible parasites along with the patient's travel history, we focused on protozoal infection as an explanation of his presentation. As noted above, serologic testing confirmed babesiosis as the cause of his unusual TTP-like presentation.

Microangiopathic hemolytic anemia (MAHA) is a non-immune intravascular hemolytic anemia characterized by the presence of schistocytes on the peripheral blood smear [1]. Thrombotic thrombocytopenic purpura (TTP), a medical emergency and an almost uniformly fatal disease without appropriate treatment, can present with MAHA [2]. The prognosis of TTP is correlated to the time from the first clinical manifestation of the TTP to the onset of plasma exchange (PEX) therapy. Recent recommendations have stated the importance of prompt initiation of PEX therapy in suspected TTP cases [3]. However, it is essential to consider other systemic disorders associated with MAHA and thrombocytopenia before initiating PEX. In our case, the patient's residence and peripheral blood smear report led to the diagnosis of babesiosis instead of TTP, which was later confirmed by Babesia DNA real-time PCR.

Babesiosis is a zoonotic parasitic infection caused by protozoa of the genus Babesia [5]. The first human babesiosis case was identified near Zagreb, Croatia in 1957 [6]. Since then, its epidemiology evolved from isolated incidents to specific endemic areas. Human Babesia species are Babesia microti, Babesia divergens, and Babesia venatorum. Among them, Babesia microti is the primary agent of most human babesiosis in the United States [3, 5, 7]. It is spread by Ixodes scapularis ticks, also known as black-legged ticks or deer ticks, mainly in the Northeastern and upper Midwest states, predominantly in New England, Connecticut, Massachusetts, Minnesota, New Jersey, New York, Rhode Island, and Wisconsin [3, 8].

The clinical manifestation ranges from asymptomatic subclinical infection to severe fatal infection depending on the host immune status and the Babesia species. In mild to moderate babesiosis, patients usually experience a gradual onset of fatigue and malaise with fever. Signs and symptoms like abdominal pain, nausea, vomiting, photophobia, and jaundice appear less common. Physical examination may reveal fever, icterus, hepatosplenomegaly, but no lymphadenopathy. Severe infection can occur in splenectomized and immunocompromised patients. Moreover, Babesia can relapse within a few days or a week after discontinuation of antibiotics, and the organism can persist in the body despite appropriate treatment, especially in patients with B cell lymphoma or other conditions treated with rituximab, patients with malignancy who also are asplenic, patients with organ or stem cell transplantation, and HIV/AIDS patients [7, 8]. Our patient's age and underlying comorbidities may have contributed to his development of severe babesiosis.

Other Ixodes-borne pathogens are Borrelia burgdorferi, Anaplasma phagocytophilum, Borrelia miyamotoi, Borrelia mayonii, Powassan virus, and Ehrlichia muris-like agent. In the United States, the most frequent pathogens transmitted by I. scapularis ticks are B. burgdorferi sensu stricto, B. microti, and A. phagocytophilum, the causative agent of human granulocytic anaplasmosis (HGA) [9]. Therefore, coinfection with other Ixodes-borne infection needs to be considered for any patient who experiences an illness more severe than expected and does not respond well to recommended antibiotic therapy [10].

Diagnosis of babesiosis includes identification of the organisms on thin blood smear stained by Wright and Giemsa stains. Intraerythrocytic ring form seen on the Giemsa-Wright stain has similarities with Plasmodium, a malaria-causing protozoa, which can be distinguished by careful observation. Babesia merozoites display tetrads or Maltese cross, which is rare but pathognomonic for babesiosis. Besides, extracellular merozoites and hemozoin deposit in RBC are only seen in Babesia [7, 11]. Babesia DNA real-time PCR is useful in species identification and parasite quantification [7, 12]. Babesia DNA may be detectable by PCR for several months after completing antibiotic therapy and symptom resolution. Parasite DNA has been detected up to 404 days in untreated asymptomatic patients and up to 26 months in immunosuppressive patients [13]. Serology may be used for diagnostic confirmation of babesiosis and species identification [14]. Interpreting serology is challenging to distinguish current from past infection [7]. IgM antibody is usually detected two weeks after onset of illness. A positive IgM titer is suggestive of recent infection but must be accompanied by a positive IgG titer to establish a diagnosis [7, 15]. A fourfold rise in Babesia IgG titer in acute and convalescent sera confirms recent infection, but a single positive antibody titer often cannot distinguish current from recent or past infection. The IgG titers usually exceed 1:1024 during the acute phase but typically decline to ≤1:64 within 6-12 months [12].

Treatment of mild to moderate babesiosis typically includes a combination of oral atovaquone and oral azithromycin for 7-10 days [16]. An alternative option is oral Clindamycin plus oral quinine for the same duration [17]. Symptoms typically resolve within a day or two of initiating therapy, and complete clearance of the infection occurs within three months. The optimal approach for the treatment of severe babesiosis patients consists of either intravenous azithromycin plus oral atovaquone or intravenous Clindamycin plus oral quinine [7]. These patients may require partial or complete erythrocyte exchange transfusion [7, 18].

## Conclusions

As the incidence of Babesia infection has been increasing across North America in recent years and as its clinical presentation of microangiopathic hemolytic anemia (MAHA) with thrombocytopenia is similar to other severe systemic disorders, it is crucial to be able to recognize all spectrums of babesiosis including the possibility of coinfection with other tick-borne diseases to prevent delays in diagnosis and treatment.
